# LdrB Toxin with In Vitro and In Vivo Antitumor Activity as a Potential Tool for Cancer Gene Therapy

**DOI:** 10.3390/cancers11071016

**Published:** 2019-07-20

**Authors:** Yaiza Jiménez-Martínez, Carmen Griñán-Lisón, Hoda Khaldy, Ana Martín, Alba Cambrils, Andrea Ibáñez Grau, Gema Jiménez, Juan A. Marchal, Houria Boulaiz

**Affiliations:** 1 Biopathology and Regenerative Medicine Institute (IBIMER), Centre for Biomedical Research, University of Granada, E-18100 Granada, Spain; 2 Department of Human Anatomy and Embryology, Faculty of Medicine, University of Granada, E-18012 Granada, Spain; 3 Instituto de Investigación Biosanitaria ibs.GRANADA, University Hospitals of Granada-University of Granada, 18012 Granada, Spain; 4 Research Unit “Modeling Nature” (MNat), University of Granada, 18016 Granada, Spain; 5 Fundamental Biology Service, Scientific Instrument Center, University of Granada, 18071 Granada, Spain

**Keywords:** suicide gene therapy, *ldrB* gene, colorectal cancer, breast cancer, apoptosis, cell cycle arrest

## Abstract

Due to the high prevalence of cancer in recent years, it is necessary to develop new and more effective therapies that produce fewer side effects. Development of gene therapy for cancer based on the use of suicide genes that can damage the tumor cell, without requiring a prodrug for its lethal effect, is one of the recent foci of gene therapy strategies. We evaluated the cytotoxic impact of the LdrB toxin from *Escherichia coli* k12 as a possible tool for cancer gene therapy. For that, colorectal and breast cancer cells were transfected under the control of a TRE3G promoter inducible by doxycycline. Our results showed that *ldrB* gene expression induced a drastic inhibition of proliferation in vitro, in both 2D and 3D experimental models. Moreover, unlike conventional chemotherapy, the *ldrB* gene induced a severe loss of proliferation in vivo without any side effects in our animal model. This antitumor outcome was modulated by cell cycle arrest in the G0/G1 phase and apoptotic death. Scanning electronic microscopy demonstrates that the LdrB toxin conserves its pore-forming ability in HCT-116 cells as in *E. coli* k12. Taken together, our results provide, for the first time, a proof of concept of the antitumor capacity of the *ldrB* gene in colorectal and breast cancer.

## 1. Introduction

Cancer is a disease with a major impact around the world. Its incidence is on the rise and predictions suggest that by 2030, 13 million people will die from cancer each year [1]. Currently, colorectal cancer (CRC) and breast cancer are among the cancers with the highest incidence and mortality rates. In fact, CRC is known to be the third type of cancer in incidence and the second in terms of death for both sexes combined, while breast cancer is the first type of cancer both in incidence and mortality for females [2]. On the therapeutic front, conventional treatments such as chemotherapy, radiotherapy, surgery, and hormone therapy have certain limitations [3]. Furthermore, patients undergoing current systemic therapies will suffer multiple side effects, from nausea to infertility, and develop drug resistance that leads to a considerable decrease in the therapeutic efficacy of anticancer agents [4,5]. Therefore, research for new and more efficient therapies is required. Technologies for gene transfer to tumor cells for therapeutic purposes seem to be a good option [6].

One potential approach involves the genetic modification of tumor cells by the transfer of suicide genes [7]. Suicide gene therapy can be divided into two categories: indirect gene therapy using an enzyme activating prodrug that allows the conversion of a nontoxic prodrug into a drug that is lethal in tumor cells; and direct gene therapy using toxin genes expressing toxic molecules that can affect stability of the cell membrane and reduce the viability of tumor cells [8].

Most of the suicide gene strategies developed focus on the prodrug/drug system, where the herpes simplex virus thymidine kinase gene (HSV-TK) with ganciclovir (GCV) together with the cytosine deaminase (CD) (enzyme found in bacteria and fungi, but absent in mammalian cells) with 5-fluorocytosine (5-FC) systems are the most used [9]. However, these systems have several limitations related to the limited bioavailability of the prodrug and the targeting of only rapidly dividing cells by disrupting the DNA synthesis. That makes the use of toxins, which do not require a prodrug for activation and have the ability to kill even quiescent tumor cells, attractive [10,11]. Several toxins from plants, viruses, and bacteria have been studied for antitumor suicide gene therapy [10,12,13]. The potent anti-tumor effect of the diphtheria toxin ricin, derived from plants, and pseudomonas exotoxin has been well analyzed both in vitro and in vivo [14,15,16]. The selective antitumor toxicity of apoptin, a small protein encoded by chicken anemia virus, has been revealed in a variety of tumors (i.e., cancers of prostate, breast, stomach, colon, cervix, and lung, among others) and could be used to induce apoptosis in cancer cells [11,17]. The *gef* gene, expressed in *Escherichia coli*, is able to induce apoptosis, cell cycle arrest, and the apparition of morphologic changes in a wide range of human cancer cells [12,18,19,20]. In this context, almost all bacteria contain several toxin–antitoxin (TA) modules. Toxin products target various cellular functions regulating cell proliferation and death. These toxins are usually co-expressed with their cognate antitoxins in operons called toxin–antitoxin (TA) modules [21]. One of the genes of the operon codifies a stable toxin and another gene encodes an unstable antitoxin, with frequent overlapping of both loci. Under normal growth conditions, the modules form stable complexes. Nevertheless, the proteases generated by stress favor the elimination of unstable protein antitoxins, releasing free toxins to obstruct numerous cellular functions [22,23]. The *Escherichia coli* K-12 genome encodes at least 36 putative TA systems and one of them is the *ldr/rdl* gene family. Four copies of long direct repeat (A, B, C, and D) sequences were detected upon completion of the *E. coli* genomic sequence. The *ldrB* gene encodes a small toxic protein whose overexpression leads to rapid host cell killing. The overexpression of this gene product leads to nucleoid condensation with the appearance of filled spheres and a strong inhibition of transcription and translation, resulting in a severe loss of cell viability [24,25]. The physiological function of the LdrB peptide involved in the phenotype is at present unknown; however, microarray analysis suggests that overexpression of *ldrD,* another member of the LDR family similar to the *ldrB* gene, leads to physiological alteration in purine metabolism in *E. coli* [24]

Based on these observations, we selected the *ldrB* gene to study its behavior in eukaryotic cells, since it could be a potential new candidate for use as an antitumor suicide gene. The main aim of the present study was to investigate the therapeutic potential of the *ldrB* gene using an HCT-116 colorectal carcinoma and caspase-3 deficient MCF-7 breast cancer human cell lines both in vitro and in vivo. For that, we developed new HCT-116 and MCF-7 cell lines transfected with the *ldrB* gene through the Inducible Expression System Tet-On 3G. Our results demonstrate that the *ldrB* gene causes a drastic inhibition of cell proliferation and induces apoptosis in transfected HCT-116 cells, manifesting cytotoxic activity in both tumoral models that can be used in the future as an anticancer treatment.

## 2. Results

### 2.1. ldrB Gene Expression Induces Significant Inhibition of Proliferation in HCT-116 and MCF-7 Cells

**Figure 1:**
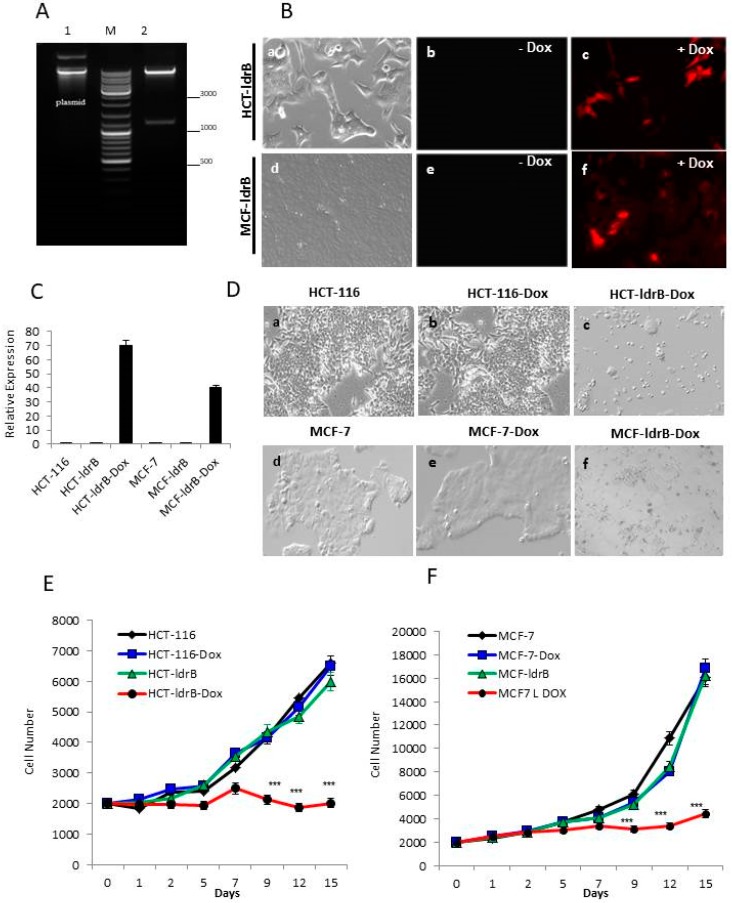
HCT-116 and MCF-7 cells stably express the *ldrB* gene after transfection. (**A**) The plasmid pLVX-TRE3G-mCherry-IdrB was identified by XhoI and ApaI digestion. M: DNA Ladder 10,000; 1, undigested circular plasmid; 2, plasmid digested with XhoI and ApaI (expected Size: 1187+8K). (**B**) HCT-116 and MCF-7 cells were transfected with the *ldrB* gene using Tet-On 3G Inducible Expression System (with mCherry). Stable, transfected cells colored red were selected. In the absence of Dox, protein fluorescence is not observed. After 24 h of Dox administration, red fluorescence emitted by m-cherry through LdrB toxin expression emerged (a, b, and c 20× and e, f, and g 10×). (**C**) Total RNA was extracted from HCT-116, MCF-7, HCT-ldrB, and MCF-ldrB cells. After 24 h of dox induction, *ldrB* expression levels were analyzed with qRT-PCR. (**D**) Effects of the *ldrB* gene on HCT-116 and MCF-7 cell lines (a) HCT-116 cells (b) HCT-116-Dox, and (c) HCT-ldrB-Dox after 15 d of culture; (d) MCF-7 cells, (e) MCF-7-Dox, and (f) MCF-ldrB-Dox after 15 d of culture (a, b, c, d, e, f, and g 10×). (**E**) Determination of the effects of *ldrB* gene expression on HCT-116 and (**F**) MCF-7 cell proliferation. Cells were cultured for 15 d to determine the growth rate. Data were obtained by an EnSight system with well imaging technology. Values represent means ± SD of quadruplicate cultures (*** *p* < 0.001).

To study the effect of the *ldrB* gene in HCT-116 and MCF-7 cells, a Tet-On 3G Inducible Expression System with mCherry was used. Cells were transfected by pCMV-Tet3G and pLVX-TRE3G-mCherry containing the *ldrB* sequence (Figure 1A). This system allows the simultaneous expression of *ldrB* gene and a red fluorescent protein (mCherry) marker, under the control of a TRE3G promoter (PTRE3G) that binds the Tet-On 3G transactivator protein in the presence of Dox.

After 24 h of HCT-ldrB and MCF-ldrB cell Dox (2 µg/mL) induction, detection of the expression of the *ldrB* gene was studied by fluorescence microscopy through the fluorophore m-Cherry, which is found in the PTRE3G promoter, and its expression therefore acts as an indicator of the expression of the gene of interest by producing a red protein. The *ldrB* gene was expressed in HCT-ldrB and MCF-ldrB cells cultured with Dox, but not in those without Dox (Figure 1B). The presence of mRNA from the ldrB gene in HCT-ldrB-Dox and MCF-ldrB-Dox cells was verified by qRT-PCR (Figure 1C).

In 2D culture, we found no significant differences in growth patterns such as morphology, cell size, and growth rate between HCT-116 and MCF-7 control cells with or without Dox treatment and HCT-ldrB and MCF-ldrB cells transfected without Dox induction. These control cell lines were characterized by an exponential increase in the proliferation rate (Figure 1D–F).

In the HCT-ldrB cell line, inhibition of the proliferation rate was observed when Dox was administered (Figure 1D,E). Thus, HCT-ldrB-Dox cells suffered a slight inhibition of the proliferation (19.28%) on the five first days of induction with Dox. This inhibition increased, reaching 49.18%, 65.44%, and 69.35% after 9, 12, and 15 d of induction, respectively, compared to the HCT-116 control cells (Figure 1D). The same trend was observed in MCF-ldrB-Dox cell line with inhibition reaching 48.92%, 68.97%, and 72.76% after 9, 12, and 15 d of induction, respectively, compared to the MCF-7 control cells (Figure 1F). These results derived from cell count evolution using the EnSight^™^ multimode plate reader combined with workflow-based Kaleido™ data acquisition and analysis software, and results have been confirmed by the analysis of cell confluence. 

**Figure 2:**
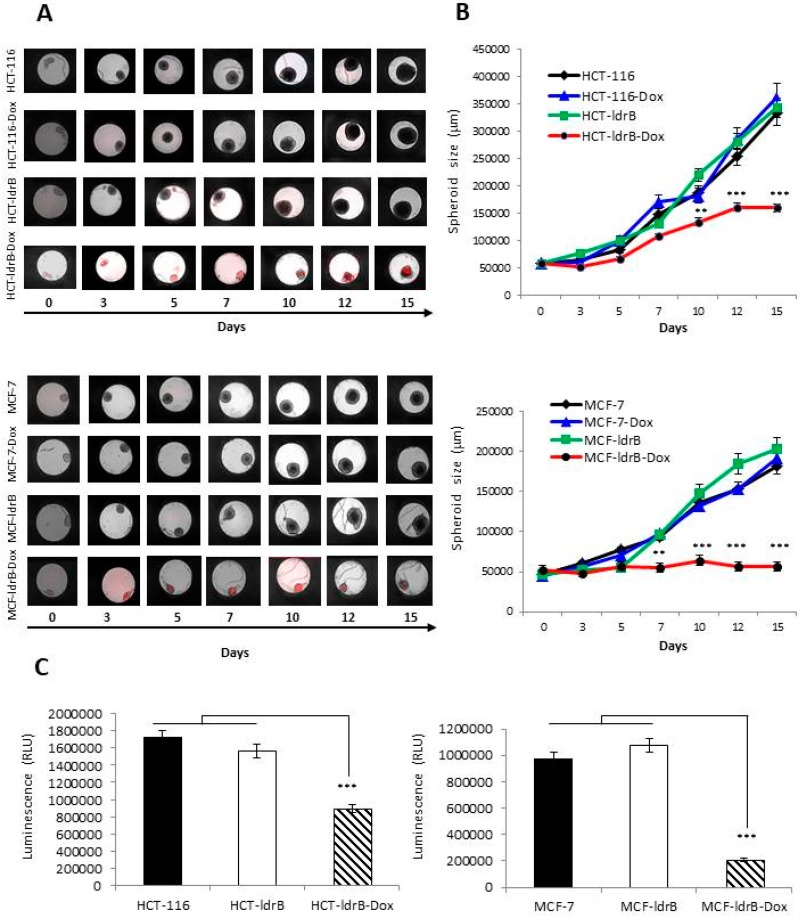
*ldrB* gene expression induces inhibition of HCT-116 and MCF-7 spheroid proliferation. (**A**) HCT-116, HCT-116-Dox, HCT-ldrB, HCT-ldrB-Dox, MCF-7, MCF-7-Dox, MCF-ldrB, and MCF-ldrB-Dox spheroids were cultured for 15 d to determine the growth rate. Brightfield and fluorescence overlay images were obtained by an EnSight system with well imaging technology. (**B**) Size of HCT-116, HCT-ldrB, MCF-7, and MCF-ldrB spheroids at different days (0, 3, 5, 7, 10, 12, and 15) of gene induction. (**C**) ATPlite level of HCT-116, HCT-ldrB, and HCT-ldrB-Dox assessed by ATPlite-3D assay kit. Values represent means ± SD of quadruplicate cultures (*** *p* < 0.001 vs. control).

Proliferation rate was also determined on HCT-116, HCT-ldrB, MCF-7, and MCF-ldrB cell lines in a 3D culture model, both in the presence and absence of Dox. Images were obtained (Figure 2A), and later, the perimeter was calculated to identify the size of the spheroid (Figure 2B).

The proliferation study demonstrated a significant growth inhibition of the HCT-ldrB-Dox spheroids compared to control cells. After 7 d of *ldrB* gene expression, the size of the spheroids in colorectal cancer HCT-ldrB-Dox were reduced compared to the control ones, being more significant after 10, 12, and 15 d postinduction. Results obtained showed that on days 3 and 5 of gene expression there was 20% inhibition of proliferation. This inhibition increased with time, reaching 29.19%, 37.39%, and 52.67% after 10, 12, and 15 d of treatment. In the case of the MCF-7 line, while the spheroids of the control cell lines began to proliferate exponentially, the spheroids of the MCF-ldrB-Dox cell line stopped maintaining their initial size. Thus, inhibition of the proliferation of the MCF-7-ldrB-Dox cell line increased over time, reaching 58.45%, 67.22%, and 72.52% after 10, 12, and 15 d of treatment, respectively. This inhibition was in concordance with ATPlite level decrease observed at the end of the experiments by ATP lite assay kit (Figure 2C).

### 2.2. ldrB Produces G0/G1 Arrest and Apoptosis in HCT-116 and MCF-7 cells

**Figure 3:**
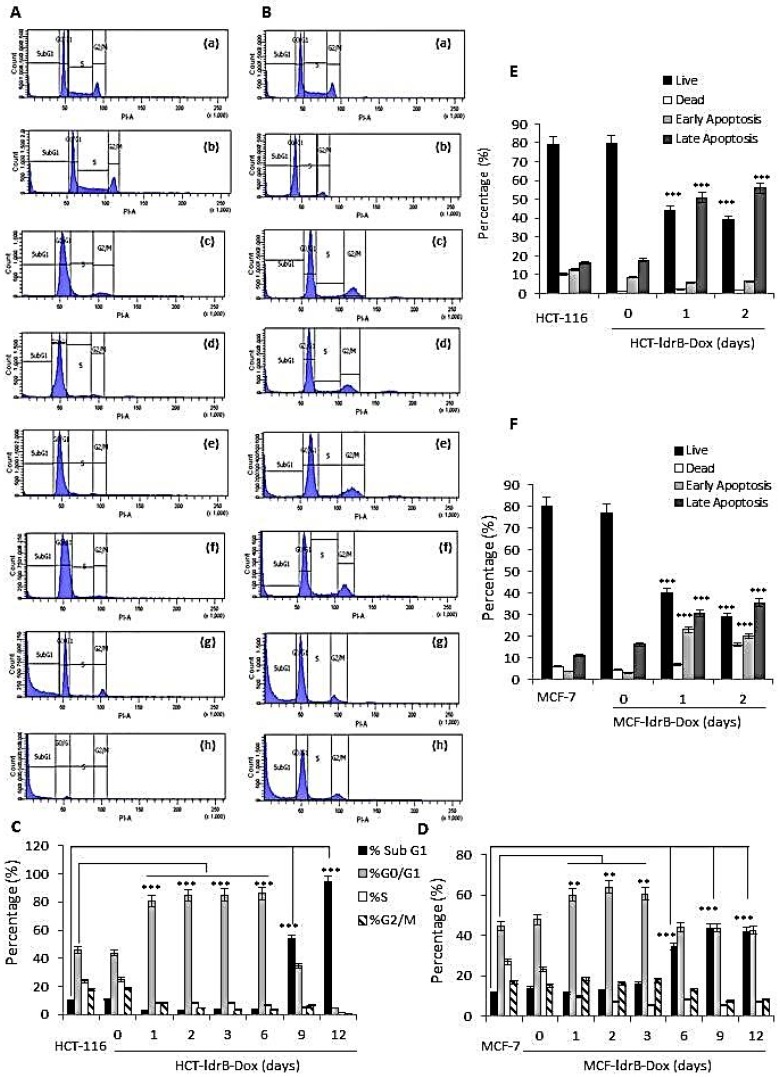
*ldrB* gene induced cell cycle arrest in G0/G1 and apoptosis in both HCT-116 and MCF-7 cell lines. (**A**) Cell cycle analysis of HCT-116 cells treated with the *ldrB* gene after staining with propidium iodide. (a) HCT-116, (b) HCT-ldrB, and (c)–(h) HCT-ldrB after 1, 2, 3, 6, 9, and 12 d of Dox induction, respectively. (**B**) Cell cycle analysis of MCF-7 cells treated with the *ldrB* gene after staining with propidium iodide. (a) MCF-7, (b) MCF-ldrB, and (c)–(h) MCF-ldrB after 1, 2, 3, 6, 9, and 12 d of Dox induction, respectively. (**C**) Percentage of HCT-116 and HCT-ldrB-Dox cells in sub-G1, G0/G1, S, and G2/M phases. (**D**) Percentage of MCF-7 and MCF-ldrB-Dox cells in sub-G1, G0/G1, S, and G2/M phases. (**E**) Late apoptosis is enhanced by *ldrB* gene expression in HCT-116 and (**F**) MCF-7 cells. Data are expressed as the mean ± SD of the mean of three independent experiments (** *p* < 0.01 vs. control and *** *p* < 0.001 vs. control).

We used flow cytometry to examine the cell cycle distribution (Figure 3A,B) depending on DNA content, which discriminates between G0/G1, S, and G2/M cell phases. HCT-ldrB and MCF-ldrB cells were induced at different time-points with Dox for 12 d. 

HCT-116 cell line, used as control, contained 45.6% G0/G1 cells, 24% S phase cells, and 17.6% G2/M phase cells (Figure 3A,C). At 24 h after *ldrB* expression, HCT-ldrB cells showed an increase in the G0/G1 phase population (80.7%) and a decrease in the number of S phase (8.35%) and G2/M phase cells (8.33%). This trend was maintained until day 9, when we observed a large accumulation in the phase sub-G1 (53.8%), reaching 94.01% on day 12. Furthermore, the MCF-7 control cell line contained 44.7% G0/G1 cells, 27% S phase cells, and 17% G2/M phase cells (Figure 3B,D). At 24 h after *ldrB* expression, MCF-ldrB cells showed an increase in the G0/G1 phase population (60%) and a decrease in the number of S phase (9.8%), while G2/M phase cells had hardly been modified (18.6%). This trend was maintained until day 6, when we observed a large accumulation in the phase sub-G1 (34.5%), reaching 41.8% on day 12. These results may reflect that, by using this methodology, a critical concentration of LdrB toxin is needed to provoke cell sub-G1 accumulation detection.

Having seen that the sub-G1 peak did not appear until day 9, we wanted to analyze apoptosis by annexin V and propidium iodide (PI), a more sensitive technique. HCT-ldrB and MCF-ldrB showed no significant modification in relation to HCT-116 and MCF-7 control culture. However, our results showed that in both HCT-ldrB-Dox and MCF-ldrB-Dox, an increase of early and late apoptotic cells that reached late apoptosis over time were 50.93% and 30.5% by 1 day and 62.02% and 35.3% by 2 days post-Dox induction, respectively (Figure 3E,F).

### 2.3. Morphological Findings. 

**Figure 4:**
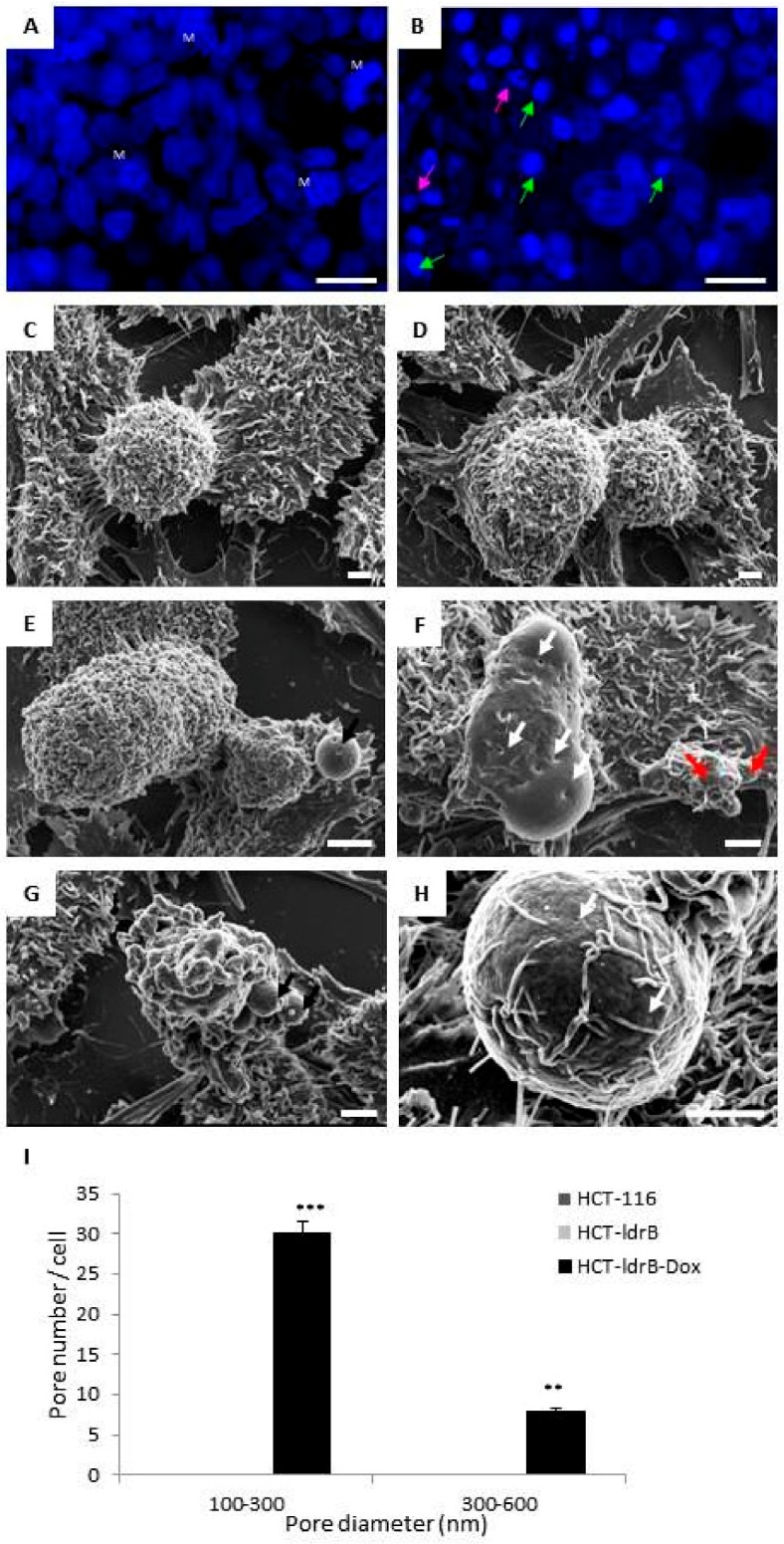
Expression of the *ldrB* gene induces apoptosis and pore formation in HCT-ldrB-dox cells. (**A**) Hoechst 33342 staining of HCT-116 cells showed a uniform nuclear stain and high levels of mitosis (M), while in HCT-ldrB-dox cell line (**B**), a large number of cells with nuclear fragmentation (purple arrow), many with condensed chromatin (green arrow), was observed (scale bar = 20 μm). Scanning electron microscopic images of HCT-116 (**C**) and HCT-ldrB (**D**) control cells were rounded and adhered to the culture surface with multiple filipodia. HCT-ldrB-Dox cell line showed cells of different sizes with signs of apoptosis with cytoplasmic membrane disruption, many apoptotic bodies, and clear signs of substrate detachment (**E**–**G**). Most of the cells had pores on their membrane surface (**E,F,H**). White arrows signal apoptotic bodies, and black arrows signal pores (scale bar = 2 μm). (I) Quantification of pore number. The height of each bar represents the number of pores with diameters in the interval between its lower and upper bounds on the *x*-axis. Pore diameters between 100–600 nm could be observed only in HCT-ldrB-Dox cells. Quantifications were measured from the SEM images (8 random photos) from three independent sample preparations. The data are represented as mean ± SD (** *p* < 0.01 vs. control and *** *p* < 0.001 vs. control).

Hoechst 33342 staining of HCT-116 cells (Figure 4A) showed a uniform nuclear staining and high levels of mitosis (M), while in the HCT-ldrB-dox cell line (B) we found a large number of cells with nuclear fragmentation (purple arrow) and many with condensed chromatin (green arrow), both being signs of apoptosis. Observations of HCT-116 and HCT-ldrB cells with SEM showed rounded cells covered by multiple microvillous extensions and strongly adhered to the culture flask surface (Figure 4A,B). The induction of *ldrB* gene expression in the HCT-ldrB cell line showed cells of different sizes and multiple morphological changes. In fact, in the same space we could observe giant cells with the unstructured cytoplasmic membrane next to dwarf cells manifesting apoptotic bodies (Figure 4C–E). Moreover, transfected cells showed a “flat cell surface” due to the loss in microvilli and filopodia structures compared to controls (Figure 4C–F). Interestingly, the cytoplasmic membrane of most of the cells showed multiple “pores” of different sizes that were never detected in the control lines (Figure 4C,F).

### 2.4. The ldrB Gene Inhibits Growth of Tumor Xenografts in Nude Mice without Induction of Subacute Toxicity

**Figure 5:**
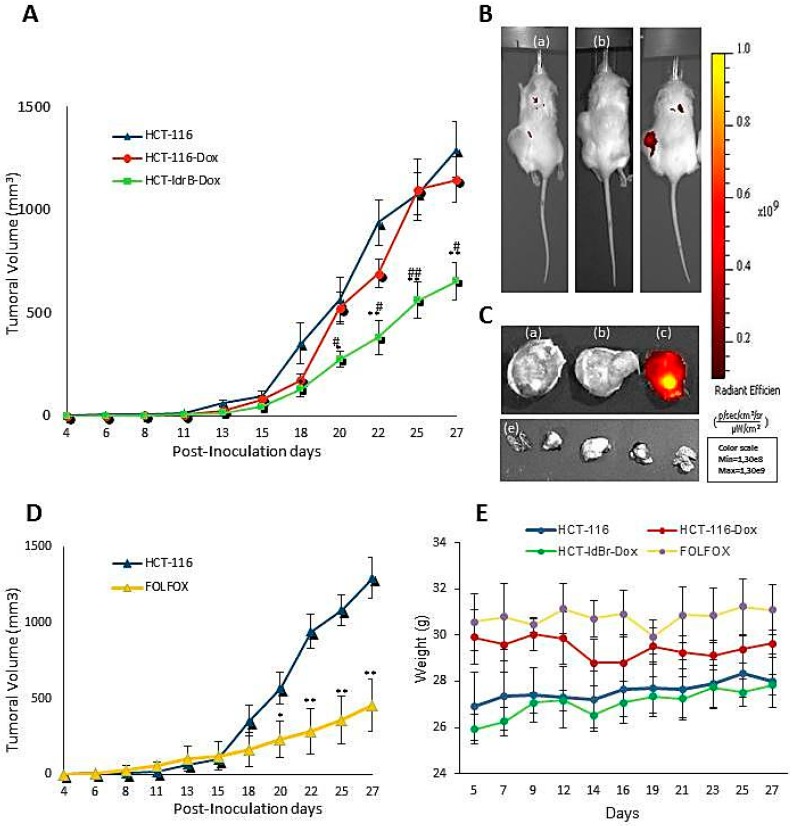
*ldrB* gene expression in HCT-116 cells reduces tumor size in vivo. (**A**) Growth rates of HCT-ldrB tumor xenografts in SCID mice (bars indicate standard deviation of tumor volumes, *n* = 8 sites injected, *p < 0.05, ** *p* < 0.01 HCT-116 and HCT-ldrB-Dox; # *p* < 0.05, ## *p* < 0.01 HCT-116-Dox and HCT-ldrB-Dox). (**B**) In vivo imaging of tumor-bearing mice (a) HCT-116, (b) HCT-116-dox, and (c) HCT-ldrB-Dox. (**C**) Representative images of fluorescent signal of (a) HCT-116, (b) HCT-116-dox, and (c) HCT-ldrB-Dox resected tumors. (e) Images of mouse organs treated with the *ldrB* gene (HCT-ldrB-Dox) free of metastasis. All images were captured by an IVIS Lumina Imaging System after 27 d of treatment. (**D**) Growth rates of tumor xenografts treated by FOLFOX in SCID mice (bars indicate standard deviation of tumor volumes, *n* = 8 sites injected), and (**E**) changes in the body weights of HCT-116, HCT-116-Dox, HCT-ldrB-Dox, and FOLFOX groups of mice during the treatment period.

Following the criteria and recommendations of the ethics committee of our organization, we performed the in vivo tests only with colon cancer lines in order to minimize the number of mice used, thus avoiding unnecessary animal suffering. We first evaluated the therapeutic efficacy of *ldrB*-mediated suicide gene therapy using heterotopic tumor xenografts established by HCT-116 and HCT-ldrB cell lines (Figure 5A). Parental HCT-116 cells and HCT-ldrB cells were subcutaneously injected in BALB/c mice. Once tumors were palpated, we proceeded to the administration of Dox (100 mg/kg) by intraperitoneal injection, setting that day as day 0. A third group of mice bearing HCT-116 tumors without the administration of Dox was used as a control. Our results demonstrated that Dox by itself had no effect on tumor proliferation. Thus, we did not observe significant differences in the growth of HCT-116 and HCT-116-Dox tumors. However, proliferation of HCT-ldrB-Dox tumors was significantly inhibited by *ldrB* gene expression after 18 d (62.45%, Figure 5A). This antitumor effect remained until the end of the experiment, maintaining an average tumor volume of around 50% lower in comparison to the control group (Figure 5A). In vivo imaging of tumor-bearing mice and resected tumors confirmed the expression of *ldrB* gene in HCT-ldrB-Dox tumors as shown by representative images of the fluorescent signal in Figure 5B,C. Furthermore, no significant difference between tumors treated with *ldrB* gene and FOLFOX was detected. Mice treated with FOLFOX showed a strong inhibition of tumor growth, reaching 64.94% at the end of the experiment (Figure 5D). Moreover, mice with HCT-ldrB-Dox tumors presented no weight loss or unusual behavior (Figure 5E), and we did not find any detectable toxicity or metastasis in the liver, lung, heart, or kidneys (Figure 5C (e)). These data indicate that the *ldrB* gene did not cause any systemic damage.

## 3. Discussion

Development of gene therapy for cancer based on suicide genes that can directly damage the tumor cells, without requiring a prodrug for its lethal effect, is one of the purposes of gene therapy strategies. In this context, in the present manuscript, we demonstrate for the first time that expression of the *ldrB* gene induced a drastic inhibition of cell proliferation in colorectal and breast cancer both in vitro and in vivo. In our construction, the *ldrB* gene is under the control of Tet-On 3G technology, a doxycycline-based system that can be controlled in quantity and time. In the presence of Dox, a Tet-On 3G transactivator promoter (transcriptional regulator) undergoes a conformational change to bind specifically to tet operator sequences located in a PTRE3G promoter, which ultimately activates the transcription of the LdrB toxin. The inducible promoter PTRE3G provides very low basal expression and high maximal expression after induction [26]. Thence, gene expression can be suppressed in the absence of Dox. Also, another advantage provided by this system is the low toxicity derived by tetracyclines, for example doxycycline, which does not produce any adverse effects [27,28] with doses at these antibiotic concentrations. Thus, our system does not need prodrug administration, which avoids possible problems related to general toxicity, drug release, and bioavailability found in indirect gene therapies like HSV-TK/GCV and CD/5-FC. Moreover, our system, by expressing fluorescence, can allow us to track the tumor cells in case of metastasis; hence, it will have a theranostic function that makes it highly promising for further application in the clinic.

For our in vitro study, the technology used to study cell proliferation is part of the new imaging methods offered by the EnSight system. This imaging technology is an ideal instrument for determining important cytometric parameters, such as cell numbers and confluence levels, without the use of fluorescent dyes, which can sometimes be toxic over long incubation periods. Furthermore, with a fast image time (<5 min per plate), data are obtained with the least disturbance possible to the cells. This technology is based on brightfield imaging, which presents a nondestructive readout, allowing the collection of data on the kinetics of possible toxic effects. We used both 2D and 3D cultures to determine the effect of the *ldrB* gene. In both experimental models, LdrB toxin was able to induce a drastic inhibition of cell proliferation in colorectal and breast human cancer cell lines. For the MCF-7 cell line, about 70% of growth rate inhibition was observed in both 2D and 3D models. Moreover, *ldrB* gene expression induces a total blockage on spheroid proliferation. However, for the colorectal cancer cell line, while a 69.36% rate of growth inhibition was observed in 2D culture, the diameter of the tumor spheroid in 3D culture was reduced to 52.67%. Bacterial toxins can kill cells or reduce their number using different processes that control proliferation, apoptosis, and differentiation [29]. The ATPlite assay results were in concordance with the diameter of the spheroid, suggesting a decline in the viability of these cells. Monitoring ATP is a good indicator of cytostatic, cytocidal, and proliferation effects because ATP concentration decays rapidly when cells undergo apoptosis or necrosis.

Based on these results, we selected the colorectal cancer line to perform the in vivo assay and to analyze the mechanism of action of *ldrB* gene. The in vivo studies confirmed the results obtained in the 3D model showing a 50.73% tumor growth inhibition. This difference in the percentage of inhibition of colorectal cancer cell growth in 2D and 3D cultures could be due to that in conventional 2D conditions, since there is no stroma and tumor structural architecture, it is not possible to create a transport gradient like that of an in vivo tumor microenvironment [30]. In 2D cell cultures, the cell-to-cell and cell-to-matrix interaction, important for proliferation, differentiation, and cellular functions in vivo, is lost [31]. In contrast, 3D cultures simulate the extracellular matrix composition better than a 2D-culture [32]. In this study, a gravity ULA plate system was used for the establishment of colon spheroids. This system combined with a method for measurement of tumor size in both control and LdrB-expressing cells produced a more real outcome than 2D culture. In colorectal cancer cells, results obtained showed a growth inhibition in the cells transfected with the bacterial toxin similar to those obtained in in vivo analysis, confirming once again that cell lines grown as spheroids more closely mimic solid tumors [32]. A spheroid model is a way to get closer to the clinical situation when studying cancer drugs and radiotherapy and, therefore, avoid the suffering of animals that, ethically, we have to minimize [33]. Moreover, these results are similar to those obtained in tumors treated with the main drugs of CRC chemotherapeutics such as Fluorouracil [34] or FOLFOX [35,36] but without their numerous side effects. Moreover, Li and collaborators described that FOLFOX caused a sharp loss of body weight accompanied by swelling and anal bleeding that have forced suspension of the treatment to allow the mice to recover after only one week of treatment [35]. 

In the present work, the possible mechanism by which LdrB toxin induced cell death in HCT-116 cells was determined using flow cytometry. Our results revealed a marked accumulation in the G0/G1 phase (86.05%) of the HCT-116-LdrB-Dox cells during the six first days compared to the control (86.05%). After nine and twelve days of treatment, a more obvious sub-G1 cell cycle accumulation was observed (53.81% and 94.01%, respectively). Similar results were reported after exposure to other toxins such as posterior salivary gland toxin in MCF-7 cells [37] and LukS-PV, a pore-forming cytotoxin secreted by *Staphylococcus aureus* in human acute myeloid leukemia THP-1 cells [38], among others. 

Moreover, in concordance with these results, we found a significant increase in both early and late apoptosis for both MCF-7 and HCT-116 cells from the first day of *ldrB* gene induction. Taking into account that the sub-G1 peak did not appear until day 9, our results confirm that annexin V and IP are more sensitive for apoptosis detection. In addition, the *ldrB* gene, as a member of toxin–antitoxin (TA) systems, is known to form part of the apoptotic systems in the *E. coli* genome to regulate cell growth and death under various growth and stress conditions [23]. Reported targets of TA toxins include DNA replication, mRNA stability, ribosomes, cell division, and ATP synthesis [39]. These results were confirmed by Hoechst 33342 staining and SEM, where we found that cells exposed to *ldrB* gene presented nuclear fragmentation and condensed chromatin in addition to a flat appearance, due to the loss of microvilli and filopodia, as well as a number of apoptotic bodies. The ability of *ldrB* to induce apoptosis in colon cancer is of great interest since this type of tumor is known for its resistance to apoptosis phenomena [40,41,42]. Furthermore, in bacterial cells it is known that the *ldrB* gene is a pore-forming toxin [43]. The transmembrane domain of this toxin is expected to be localized, interacting with other proteins in the cell membrane or periplasm and/or localized in the inner membrane, where the oligomeric form of the toxin leads to pore-like structures and membrane permeabilization [25]. Consistent with the toxin effect upon the prokaryotic cell membrane, we found the same pore-forming capacity in the plasmatic membrane of HCT-116 human cells expressing the *ldrB* gene explored by SEM. This can probably result in an extensive degradation of plasma membrane and lactate dehydrogenase (LDH) release similar to that observed in many other pore-forming toxins [44].

Gene therapy has not yet successfully gained clinical significance due to the lack of targeting and tumor specificity. In this manuscript we have reported the successful use of the *ldrB* gene as an anticancer gene therapy system, both in vitro and in vivo. The great advantage of LdrB toxin is its reduced size (only 35 amino acids), hence its delivery would be much easier than other toxins that are currently in clinical trials for different types of cancer such as diphtheria A toxin (535 amino acids) and Botulinum toxin (1249 amino acids) (NCT00595088, NCT00393809 NCT01413087; NCT01822210; CT02965976). However, *ldrB* gene activity is not tumor-specific, and it will be necessary to create this specificity, as in most of the toxic genes used in gene therapy. Currently, we are working on the design of a new vector (Lentiviral vector) under the control of specific enhancer/promoter genes such as human telomerase reverse transcriptase (hTERT) overexpressed in tumor cells or carcinoma embryonic antigen (CEA) overexpressed in colon cancer cells. Tumor-specific promoters could induce a specific expression of therapeutic genes in a type of tumor increasing their localized activity, as described by Rama et al., using CEA to direct E gene expression towards colon cancer cells [45] Phael et al. also demonstrated the anticancer and selective potential of *Clostridium perfringens* enterotoxin (CPE) in vivo, using an intratumor CPE vector transfer in a patient-derived subcutaneous xenograft model derived from a metastatic colorectal tumor, that led to tumor growth inhibition [46]. Moreover, combination therapies are usually more effective than monotherapy [47], and “classical” chemo-/radiotherapy could improve the tumoral response against the *ldrB* gene. In summary, our results suggest that further investigation will be necessary to (1) assess the molecular mechanism for which HCT-116 and MCF-7 expressing the *ldrB* gene undergoes death and (2) to evaluate whether this toxin is able to induce bystander killing phenomenon. The *ldrB* gene is a suicide gene candidate with a potent antitumor effect in vivo and that, probably, will contribute to eradicating tumor mass in combination with surgery or classic radio- or chemotherapy. 

## 4. Materials and Methods 

### 4.1. Plasmid Construction and Transfection 

Tet-On^®^ 3G Inducible Expression System containing pTRE3G-mCherry and pCMV-Tet3G vectors was purchased from Clontech. The *ldrB* gene flanked by the NdeI site ant 5′ and EcoRI site at 3′ was synthesized and subcloned into pLVX-TRE3G-mCherry (Clontech, Palo Alto, CA, USA) previously digested with the same restriction enzymes. The construct was subjected to electrophoresis in 1% agarose gel. Subsequently, the band used was purified using the Gene-clean Kit (MP Biomedicals, Irvine, CA, USA). The vector and the insert were subjected to a ligation reaction of cohesive ends, which allowed proper orientation of the insert. Then, to verify the ligation between the vector and the *ldrB* gene, on one hand, a complete sequencing of the vector was performed, and, on the other hand, the construction was digested with XhoI and ApaI restriction enzymes, and an agarose gel electrophoresis was performed. The appearance of two bands corresponding to the 1187 pb and 8 kb confirmed the correct structure of our construction (Figure 1A). This construction was used to transform competent *E. coli* bacteria, which allowed us to obtain the quantities necessary for subsequent transfection experiments. 

### 4.2. Production of Stable Inducible Cell Clones 

The Tet-On^®^ 3G Inducible Expression System (with mCherry) was used. HCT-116 cells were initially transfected with pCMV-Tet3G polymer (Xfect 1.5 µL: 5 µg plasmid) (Clontech Laboratories, Inc., Mountain View, CA, USA), and successfully transfected cells were selected for geneticin (G418) (800 µg/mL) resistance. These cells were transfected with pTRE3G-mCherry-ldrB, selected for both G418 (200 µg/mL) and puromycin (1.5 µg/mL). All clones were cultured in the presence of doxycycline (Dox, 2 µg/mL, 24 h) to induce *ldrB* gene expression and red fluorescence. Then, they were selected by cell sorting by FACSan flow cytometer (Becton Dickinson, San Jose, CA, USA), with an excitation maximum of 587 nm and emission maximum of 601 nm (Clontech Laboratories, Inc. (Mountain View, CA, USA)), and cloned by limiting dilution to obtain several clones with similar characteristics; hence, we selected one to perform all the studies and we called it HCT-ldrB.

### 4.3. Cell Culture and Drug 

The HCT-116 colorectal carcinoma and MCF-7 breast cancer human cell lines were from Biobanco (Sistema Sanitario Público Andaluz, Granada, Spain). Cells were grown in Dulbecco’s modified Eagle’s medium (DMEM) (Sigma, St. Louis, MO, USA), supplemented with 10% fetal bovine serum (FBS) and 1% penicillin/streptomycin (P/S) (Sigma, St. Louis, MO, USA), under air containing 5% CO_2_ in an incubator at 37 °C.

For selection of transfected clones, we supplemented growth medium with G418 (800 µg/mL) and puromycin (1.5 µg/mL). We used a supplemented growth medium with G418 (200 µg/mL) and puromycin (0.75 µg/mL) for maintaining the selected colonies. For gene expression induction we used doxycycline (Dox, 2 µg/mL). All antibiotics were provided by Clontech Laboratories, Icn. (Mountain View, CA, USA). For cell sorting using flow cytometry, mCherry was detected by excitation with a 570 nm green/yellow solid-state laser, and the fluorescent signal was captured in a detector with a BP filter of 620 ± 20 nm.

### 4.4. Microscopy Analysis. 

Gene expression was confirmed in HCT-ldrB after 24 h of Dox induction with excitation at 587 nm. Fluorescent microscopy analysis was carried out with a Nikon Eclipse Ti (Nikon Instruments Inc., Melville, NY, USA). 

### 4.5. Real-Time Quantitative PCR (qRT-PCR). 

For quantitative real-time PCR (qRT-PCR) analysis, total RNA was extracted from HCT-116, HCT-ldrB, HCT-ldrB-Dox, MCF-7, MCF-ldrB, and MCF-ldrB-Dox cells after 48 h of induction with Dox with an RNAzol RT kit (MRCgene, Cincinnati, OH, USA), and 1 μg of RNA from each cell line was then reverse-transcribed into cDNA using a PrimeScript RT Master kit according to the manufacturer’s protocol. A qRT-PCR assay was done using SYBR Green PCR Master Mix (Promega, Madison, WI, USA)

The PCR reaction was performed in a volume of 25 μL. The PCR program was as follows: 90 °C for 2 min, 40 cycles of 95 °C for 15 s, and 60 °C for 60 s. The relative expression of each gene was calculated using the 2ΔΔ*C*_t_ method. The sequences of the forward/reverse PCR primers are as follows: forward 5′-ATATGATGACGCTCGCGCAG-3′, reverse 5′- TCTTACTTCCGGTTACGCCAC-3′.

### 4.6. Two-Dimensional Cell Proliferation Assay

HCT-116, HCT-ldrB, MCF-7, and MCF-ldrB cell lines were maintained in DMEM without red phenol (Sigma), supplemented with 10% FBS, 100 μg/mL penicillin, and 100 μg/mL streptomycin. One thousand cells were seeded onto 96-CellCarrier Plates™ (clear bottom, black). Plates were incubated at 37 °C in the presence of 5% CO_2_.

At 24 h post seeding, 2 µg/mL of Dox was added into different wells of HCT-116, HCT-ldrB, MCF-7, and MCF-ldrB, and others without Dox were maintained as a control. Every 2 d the medium was changed, and assay plate was loaded into an EnSightTM Multimode Plate Reader (PerkinElmer, Waltham, MA, USA) for imaging-based kinetic proliferation assay for up to 15 d after initiation of treatment (day 0). Both brightfield images and digital phase imaging (DPI) applying a contrast enhancement filter were acquired. Plates were kept under controlled conditions (5% CO_2_, 37 °C, and 90% humidity) between each reading. Images were automatically analyzed for cytometric parameters, such as cell number and confluency level, using predefined image analysis algorithms provided by the EnSight system’s Kaleido 2.0 Data Acquisition and Analysis Software (PerkinElmer, Waltham, MA, USA). With this new label-free method, proliferation analysis was carried out without the use of fluorescent nucleus staining, enabling long-term proliferation experiments.

### 4.7. Three-Dimensional Cell Growth Assay

Three-dimensional spheroids were generated and cultured in the InSphero GravityTRAP TM ULA 96-well plates (PerkinElmer) according to the manufacturer’s instructions. These plates were conically shaped, flat-bottom, clear 96-well polystyrene microplates, facilitating the formation of spheroids after incubating seeded cells. In addition, plates were coated to turn them into an ultr-low-attachment (‘ULA’) surface, preventing mammalian cell adherence. Five hundred cells per well were seeded, and spheroid maturation occurred within 1 or 2 d of seeding. Every 2 d the medium was changed. Spheroid growth was measured for up to 15 d after initiation of treatment (day 0) with 2–3 d intervals. Between measurements, the microtissues were incubated at 37 °C and 5% CO_2_. Spheroids were imaged using a 4× objective on the image module of an EnSight TM plate reader (PerkinElmer), and both brightfield and mCherry channels were set with NIR LED and True GREEN LED respectively. Acquired Images were automatically analyzed by Kaleido 2.0 software based on detection masks generated with a custom-developed algorithm that automatically identified and measured spheroid areas. 

### 4.8. ATPlite-Based Luminescence Assays

ATPlite™ 3D kit (PerkinElmer, Waltham, MA, USA) was used for ATPlite content endpoint measurements to confirm well imaging results. At the end of the 3D cell growth assay, spheroids were lysed, and detection reagent containing luciferase and luciferin was added according to kit instructions. Luminescence was measured using the default settings on the EnSight system.

### 4.9. Cell Cycle Analysis and Measurement of Sub-G1 Cells by Flow Cytometry

Cell cycle phases (G0/G1, S, or G2/M) were characterized according to cellular DNA content. Fluorescence dye propidium iodide (PI) (Sigma) binds with DNA strongly at a ratio of 1:1; hence, DNA contents of the cell cycle phases were reflected by varying PI fluorescent intensities (PI excited by a 488 nm solid-state laser, the fluorescent emission being captured in a detector with a band pass (BP) 585 ± 20 nm filter). Cells in monolayer culture were harvested, washed twice with phosphate-buffered saline (PBS), and fixed in 70% ice-cold ethanol at 4 °C. Cell pellets were washed twice with PBS, resuspended in a DNA extraction solution (pH = 7.8) of acetic acid (0.1 M) and sodium phosphate dibasic anhydrous (0.2 M) in PBS, and incubated for 15 min at 37 °C. Cells were pelleted and washed once again with PBS and resuspended in PI/RNase/PBS (100 mg/mL PI and 10 mg/mL RNase) solution for 30 min at 37 °C in the dark. The percentage of cells in sub-G1, G0/G1, S, and G2/M phases was determined by (fluorescence-activated cell sorting) FACSCalibur flow cytometer (BD Biosciences, Franklin Lakes, NJ, USA). 

### 4.10. Apoptosis Analysis

Cells were plated in 6-well plates, and after 24 and 48 h of Dox induction they were trypsinized and analyzed using an annexin V-fluorescein isothiocyanate detection kit (eBioscience Inc., San Diego, CA, USA) according to the manufacturer’s instructions. The samples were immediately processed using a FACSAria III flow cytometer (Becton Dickinson, BD Biosciences) from the Scientific Instrumental Center (University of Granada).

### 4.11. Hoechst Staining

After treatment, cells were stained with the nuclear dye Hoechst 33342 (1 μg/mL) in PBS at 37 °C for 15 min and then imaged by confocal microscopy using a Leica SP2 Confocal Microscope (Telstar Instrumat, Terrassa, Barcelona, Spain).

### 4.12. Scanning Electron Microscope (SEM)

The cells were grown on sterile coverslips, induced for 48 h with Dox (2 µg/mL), and treated as described by [11]. A Hitachi S-800 scanning electron microscope (Hitachi, Tokyo, Japan) was used for observations. Counting the number of pores per cell was performed using ImageJ with quantification for each cell type from 8 random photos from three independent sample preparations.

### 4.13. In Vivo Anti-Tumor Xenograft Studies

To establish xenograft tumors, eight-week-old female NOD SCID mice gamma (NOD.Cg-Prkdcscid Il2rgtm1Wjl/SzJ, NSG) were used. All procedures were approved by the Institutional Animal Care and Use Committee at the University of Granada (ethical code: 03/07/2017/086). They were housed and maintained at 20–24 °C, 50% RH, and a 10:14 h light:dark cycle with food and water ad libitum. The HCT-116 and HCT-ldrB tumors were generated by subcutaneous injections of 1.5 × 10^5^ cells in 0.05 mL matrigel and 0.05 mL DMEM medium per mouse using 26-gauge needles. When the tumors were palpable, the animals (*n* = 8 per group) were randomly assigned to control (HCT-116 and HCT-116-Dox) and treatment (HCT-ldrB-Dox) groups. The doxycycline groups were injected intraperitoneally with a concentration of 20 mg/kg three times a week for 27 d. FOLFOX (15  mg/kg fluorouracil and 20 mg/kg folinic acid each every 48 h and 10  mg/kg oxaliplatin once a week) was used as a commercial drug to compare the therapeutic efficacy of our treatment. Tumor growth was assessed using a digital caliper, and the tumor volume was calculated by the formula V = length2 × width × π/6. An in vivo imaging system (IVIS Lumina Imaging System) was used to monitor *ldrB* gene expression.

### 4.14. Statistical Analysis

Data presented in this work were performed in triplicate and compared with Student’s *t*-tests. All data are expressed as the mean ± SD. *p*-values < 0.05 (*,#), *p*-values < 0.01 (**,##), and *p* -values < 0.001 (***,###) were considered statistically significant in all cases.

## 5. Conclusions

Taken together, our results provide, for the first time, evidence of the ability of LdrB toxin to kill colorectal and breast cancer cells, both in vitro and in vivo. In addition, this antiproliferative effect was accompanied by cell cycle arrest in the G0/G1 phase, which was related to the apoptotic phenomena. Although the main mechanism of action remains unknown at present, our findings suggest that the *ldrB* gene can be considered a promising tool for the development of new antitumor strategies.
